# Anisogamy evolved with a reduced sex-determining region in volvocine green algae

**DOI:** 10.1038/s42003-018-0019-5

**Published:** 2018-03-08

**Authors:** Takashi Hamaji, Hiroko Kawai-Toyooka, Haruka Uchimura, Masahiro Suzuki, Hideki Noguchi, Yohei Minakuchi, Atsushi Toyoda, Asao Fujiyama, Shin-ya Miyagishima, James G. Umen, Hisayoshi Nozaki

**Affiliations:** 1 Department of Biological Sciences, Graduate School of Science, The University of Tokyo, Bunkyo-ku, Tokyo 113-0033 Japan; 2 Kobe University Research Center for Inland Seas, Awaji, Hyogo 656-2401 Japan; 3 Center for Genome Informatics, Joint Support-Center for Data Science Research, Research Organization of Information and Systems, Mishima, Shizuoka 411-8540 Japan; 4 Advanced Genomics Center, National Institute of Genetics, Mishima, Shizuoka 411-8540 Japan; 5 Center for Information Biology, National Institute of Genetics, Mishima, Shizuoka 411-8540 Japan; 6 Department of Cell Genetics, National Institute of Genetics, Mishima, Shizuoka 411-8540 Japan; 7 Donald Danforth Plant Science Center, 975 N Warson Rd, St. Louis, MO 63132 USA; 8 Present Address: Department of Biological Sciences, Graduate School of Science, Kyoto University, Kitashirakawa-Oiwakecho, Sakyo-ku, Kyoto 606-8502 Japan

## Abstract

Male and female gametes differing in size—anisogamy—emerged independently from isogamous ancestors in various eukaryotic lineages, although genetic bases of this emergence are still unknown. Volvocine green algae are a model lineage for investigating the transition from isogamy to anisogamy. Here we focus on two closely related volvocine genera that bracket this transition—isogamous *Yamagishiella* and anisogamous *Eudorina*. We generated de novo nuclear genome assemblies of both sexes of *Yamagishiella* and *Eudorina* to identify the dimorphic sex-determining chromosomal region or mating-type locus (*MT*) from each. In contrast to the large (>1 Mb) and complex *MT* of oogamous *Volvox*, *Yamagishiella* and *Eudorina MT* are smaller (7–268 kb) and simpler with only two sex-limited genes—the *minus*/male-limited *MID* and the *plus/*female-limited *FUS1*. No prominently dimorphic gametologs were identified in either species. Thus, the first step to anisogamy in volvocine algae presumably occurred without an increase in *MT* size and complexity.

## Abstract (web summary)

Takashi Hamaji et al. report genome assemblies for two volvocine green algae species spanning the transition from isogamy to anisogamy. Unexpectedly, they found that both species’ mating type loci (*MT*) are simple, suggesting their transition to anisogamy occurred without increased *MT* size and complexity.

## Introduction

Male–female gamete size dimorphism is a fundamental trait found in most multicellular organisms including plants, animals, fungi, brown algae, red algae, and green algae, and has been a major topic of biological sciences since Charles Darwin first wrote on the topic of sexual selection^1^. However, such dimorphism is not found in many unicellular species which typically produce morphologically identical gametes—“isogametes”. Thus, male and female gametes differing in size—known as anisogamy—emerged independently from isogamous ancestors in various eukaryotic lineages^2^. In most lineages, emergence of anisogamy was so ancient that isogamous close relatives are long extinct, making it difficult to infer the genetic bases for steps in the transition from isogamy to anisogamy and oogamy. In contrast, the entire volvocine lineage began diverging from a single-celled, isogamous *Chlamydomonas*-like ancestor ~200 MYA to give rise to genera exhibiting a range of morphological and reproductive patterns including single-celled isogamous *Chlamydomonas*, 8- or 16-celled isogamous *Gonium*, 32-celled isogamous *Yamagishiella*, 32-celled anisogamous *Eudorina*, and >500-celled oogamous *Volvox* (Supplementary Fig. 1)^3, 4^; thus providing a rich source of material for comparative evolutionary studies of sexual cycles. Furthermore, sex-determining genes and sex chromosomal regions or mating-type loci (*MT*) have been studied extensively in both isogamous and sexually dimorphic volvocine green algae^5–11^. Therefore, this green algal group represents a unique model lineage for investigating the transition from isogamy to anisogamy based on molecular genetic data.

Charlesworth^12^ predicted that anisogamy could evolve from an isogamous genetic sex-determination system with two haploid mating types if an autosomally encoded gamete cell-size-determining gene with dimorphic alleles became loosely linked to the mating locus. Under conditions where anisogamy is favored, selection would act to promote closer linkage between the cell-size locus and mating locus, with an endpoint of complete linkage disequilibrium that might be achieved through suppression of recombination. A secondary outcome of generating a region of suppressed recombination near the mating locus would be potential for the *MT* region to capture additional genes through inversions or transposition, and to retain genes or alleles that are beneficial for their respective sex^13–15^. Thus, it is predicted that anisogamous or oogamous volvocine species will have relatively complex multi-genic *MT* loci compared with *MT* loci from isogamous species, and may contain additional genes that govern gamete size and/or other sexually selected traits. A previous comparative study of the *Volvox carteri* and *Chlamydomonas reinhardtii MT* loci was consistent with the idea of massive expansion of the mating locus in *V*. *carteri* with incorporation of many new genes, including at least one dimorphic size control gene, *MAT3*^9^, a mammalian Retinoblastoma tumor suppressor (RB) homolog in the volvocine algae that regulates the cell cycle and maintains cell size in *C*. *reinhardtii*^16^. The *V*. *carteri MAT3* homolog resides within *MT* and shows extensive divergence between male and female haplotypes^9^. Although this *MAT3* allelic dimorphism was once hypothesized to be involved in establishing anisogamy^9^, a subsequent comparative analysis of the *MAT3* sequences from various isogamous, anisogamous, and oogamous volvocine organisms indicated that the extensive *MAT3* divergence in the *V*. *carteri* lineage appear to have occurred subsequent to the emergence of anisogamy, and therefore, *MAT3* dimorphism may not be directly related to the origins of male–female gamete size dimorphism in volvocine algae^17^. While *MT* sequences of *Gonium pectorale*, an isogamous volvocine alga more closely related to oogamous *V*. *carteri* than to *C*. *reinhardtii*, has been also determined^11^, the lack of information on *MT* structures at intermediate isogamous and anisogamous stages in volvocine evolution leaves open important questions about how changes in *MT* structure and gene content relate to transitions from isogamy to anisogamy. Although Geng et al.^10^ showed that the *V*. *carteri MID* gene on its own can govern key aspects of gamete dimorphism, that study did not address the evolution of structural complexity in volvocine *MT*.

**Fig. 1:**
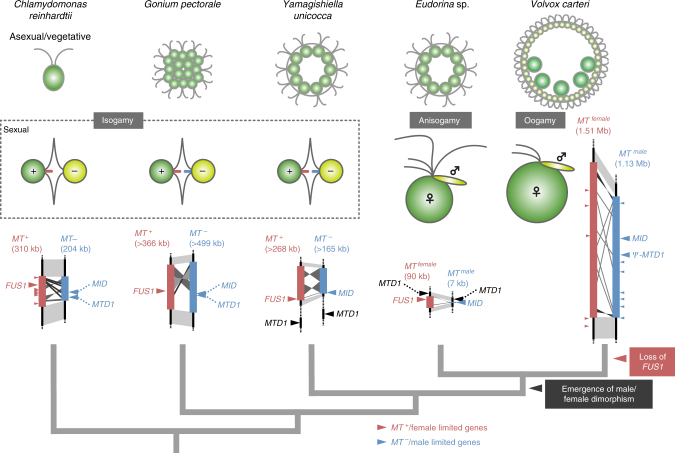
Volvocine green algal phylogeny^3, 4^ and mating-type locus (*MT*) evolution. Phylogenetic relationships of volvocine algae are illustrated with vegetative morphology, gamete morphology^18^, and *MT* structures of *Yamagishiella unicocca*, *Eudorina* sp., and three other species previously studied (*Chlamydomonas reinhardtii*^25^, *Gonium pectorale*^11^, and *Volvox carteri*^9^). The bars at the flagellar bases of the isogametes indicate tubular mating structures (TMS)^18^. In *C*. *reinhardtii*, only the *plus* gametes possess TMS (red bar). In *G*. *pectorale* and *Y*. *unicocca*, both the *plus* and *minus* gametes possess TMS (red and blue bars, respectively)

In this study, we focus on a more suitable combination for a comparative study of the transition from isogamy to anisogamy: two closely related intermediate volvocine species that bracket the transition from isogamy to anisogamy—isogamous *Yamagishiella unicocca* and anisogamous *Eudorina* sp., both of which share essentially the same vegetative (asexual) morphology (Fig. 1, Supplementary Fig. 1)^18^. We sequence the whole nuclear genomes of both mating types or sexes of *Y*. *unicocca* and *Eudorina* sp., and identify and characterize their respective *MT* regions. Contrary to expectations for increased *MT* size and gene content evolving during the transition to anisogamy, the *Eudorina* sp. male and female *MT* haplotypes are very small, 7 kb and 90 kb in size, respectively, with only two sex-specific genes and two pairs of gametologs (i.e., genes with alleles in both mating haplotypes). The highly reduced *MT* structures of *Eudorina* sp. indicates that anisogamy can evolve from isogamy without the addition of a gamete-size-control gene and without increased *MT* size and complexity.

## Results

### Mating-type loci (*MT*) of *Yamagishiella* and *Eudorina*

**Fig. 2:**
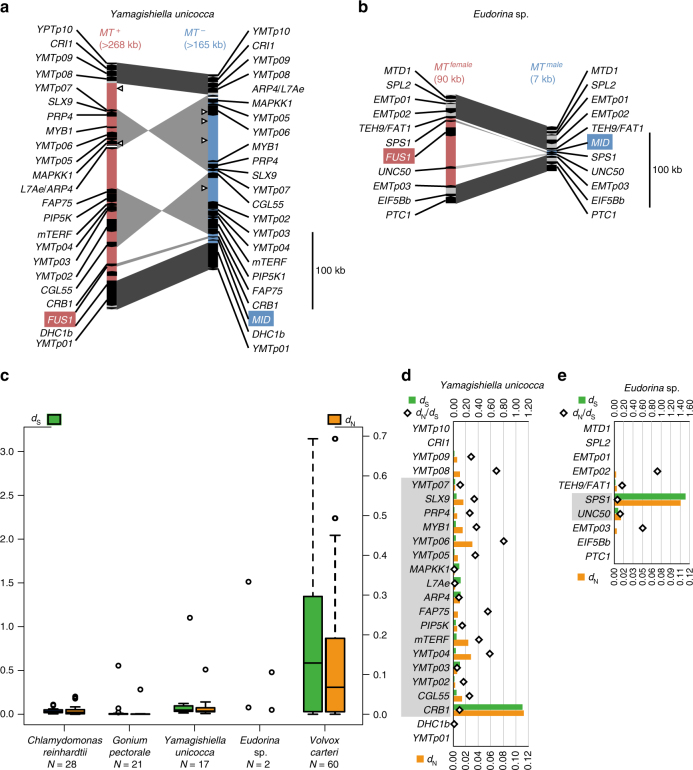
*MT* structures and molecular evolutionary analyses of *MT* gametologs in isogamous *Y*. *unicocca* and anisogamous *Eudorina* sp. **a, b** *MT* structures with sex-limited genes (*FUS1*, backed red; *MID*, blue) and gametologs of *Y*. *unicocca* (**a**) and *Eudorina* sp. (**b**) (accession nos. LC314412–LC314415). Red regions represent *plus*/female *MT*. Blue regions represent *minus*/male *MT*. Gray shading indicate a syntenic bloc. Open triangles in **a** indicate gaps between scaffolds of the de novo whole genome assembly (Supplementary Fig. 2). **c** Box-whisker plots comparing the distributions of synonymous (*d*_S_, green/left) and non-synonymous (*d*_N_, orange/right) substitution values for gametolog pairs found in rearranged regions of volvocine algal *MT* loci. Open dots are outliers from interquartile ranges except for those of *Eudorina* sp. which indicate two gametologs. **d**, **e** The *d*_N_/*d*_S_ ratios of gametologs in rearranged (gray-shaded gene names) and flanking autosomal regions of *Y*. *unicocca* (**d**) and *Eudorina* sp. (**e**) *MT*. There are no prominently dimorphic gametologs under positive selection between sexes/mating types (*d*_N_/*d*_S_ > 1)

We de novo sequenced whole genomes from both sexes of isogamous *Y. unicocca* and anisogamous *Eudorina* sp., and obtained resultant assemblies as follows: *plus* strain of *Y*. *unicocca* (2012-1026-YU-F2-6/NIES-3982, assembly “YamagishiellaPlus_1.0”), total length 134,234,618 bp of 1461 contigs with N50 of 666,310 bp; *minus* strain of *Y*. *unicocca* (2012-1026-YU-F2-1/NIES-3983, assembly “YamagishiellaMinus_1.0”), total length 140,837,241 bp of 1897 contigs with N50 of 547,037 bp; male strain of *Eudorina* sp. (2010-623-F1-E3/NIES-4100, assembly “EudorinaMale_1.0”), total length 168,620,790 bp of 2471 contigs with N50 of 377,357 bp; and female strain of *Eudorina* sp. (2010-623-F1-E8/NIES-4018, assembly “EudorinaFemale_1.0”), total length 184,032,255 bp of 3180 contigs with N50 of 564,035 bp. The resultant assemblies contained dimorphic (rearranged) haplotype regions that composed *MT* (Supplementary Figs 2 and 3). *Y*. *unicocca plus* and *minus MT* regions were 268 and 165 kb in size, respectively, which is somewhat smaller than the *MT* regions of *C. reinhardtii* and *G. pectorale* (Fig. 1, Supplementary Table 1). *Y*. *unicocca MT* included two inverted syntenic blocs of 9 and 7 collinear gametologs, and a single gene, *CRB1*, that was outside these two syntenic blocs (Fig. 2a). Two conserved sex-limited genes, *minus*-limited *MID* and *plus*-limited *FUS1* (Supplementary Figs 4–8), were also present. *Eudorina* sp. female and male *MT* haplotypes were the most highly reduced of any volvocine algal species described to date, measuring 90 and 7 kb in size, respectively (Fig. 2b, Supplementary Table 1). Only two gametologs, *SPS1* (encoding a putative spermine syntase similar to ACAULIS5 required for stem elongation in flowering plants^19^) and *UNC50* (encoding a membrane trafficking protein homolog conserved in eukaryotes^20, 21^), were found in *Eudorina* sp. *MT* (Fig. 2b). *MID* and *FUS1* homologs were also found in the male and female *MT*, respectively, as sex-limited genes. The size and GC contents of whole genome and *MT* of the two algae with the other volvocine species so far analyzed^5, 9, 11, 22–26^ are summarized in Supplementary Table 1.

Another striking finding in this study was the highly dynamic nature of *MT* haplotype structure that shows no continuity among the five volvocine *MT* regions (Fig. 1; Supplementary Data 1). Our data suggest relatively frequent turnover and haplotype reformation within *MT*, and explain the initially puzzling lack of continuity (e.g., evolutionary strata) observed between *C*. *reinhardtii* and *V. carteri MT* regions^9, 27^. Distribution of homologs of *MT* and *MT*-linked genes in the five volvocine algae (Supplementary Data 1) shows that most of these genes share the linkage to *MT*, except for *G*. *pectorale* in which majority of homologs of *MT* and *MT*-linked genes in other volvocine species are relocated to other genomic (autosomal) regions of *G*. *pectorale* as reported previously^11^. Our data further suggest that the differences in size, gene content, and structure we have observed between volvocine *MT* loci are largely uncoupled from the transition to anisogamy in this lineage.

Gametolog divergence rates for *Y*. *unicocca* and *Eudorina* sp. *MT* were compared to those of the other volvocine algae (Fig. 2c, d, e). Synonymous and non-synonymous substitutions (*d*_S_ and *d*_N_) of gametologs between mating haplotypes in these two species were smaller than those of *V*. *carteri*. Within the *Eudorina* sp. and *Y*. *unicocca* gametologs, *Eudorina* sp. *SPS1* and *Y*. *unicocca CRB1* had relatively high *d*_S_ and *d*_N_ values (Fig. 2d, e). However, the *d*_N_/*d*_S_ ratios of these two gametologs were low. Thus, we could not detect any strong patterns of selection or functional divergence between genes in the mating haplotypes of isogamous *Y*. *unicocca* and anisogamous *Eudorina* sp.

Overall, *Y*. *unicocca* and *Eudorina* sp. *MT* were smaller and simpler than *MT* regions from other volvocine species with only two conserved sex-limited genes (*MID* and *FUS1*) and no other clear examples of gametolog dimorphism. Some of the sex-limited gene homologs of oogamous *V*. *carteri* (*VcFSI1f, VcHMG1f*, and *VcMTM0097*) were conserved in the genomes of *Y*. *unicocca* and/or *Eudorina* sp. but they were autosomally encoded (Supplementary Table 2). The *MTD1* gene is a *minus*-limited gene in *C*. *reinhardtii* and *G*. *pectorale*, while in *Y*. *unicocca* and *Eudorina* sp. *MTD1* homologs are autosomal, but located nearby the *MT* region (Fig. 1, Supplementary Table 2). The presence of only two ancestral mating type genes (*MID* and *FUS1*) and lack of prominently divergent gametologs in highly reduced *Eudorina* sp. *MT* strongly disfavors the hypothesis that new sex-specific cell size-determining genes were acquired in *MT* during the emergence of anisogamy in the volvocine lineage^12, 27^.

**Fig. 3:**
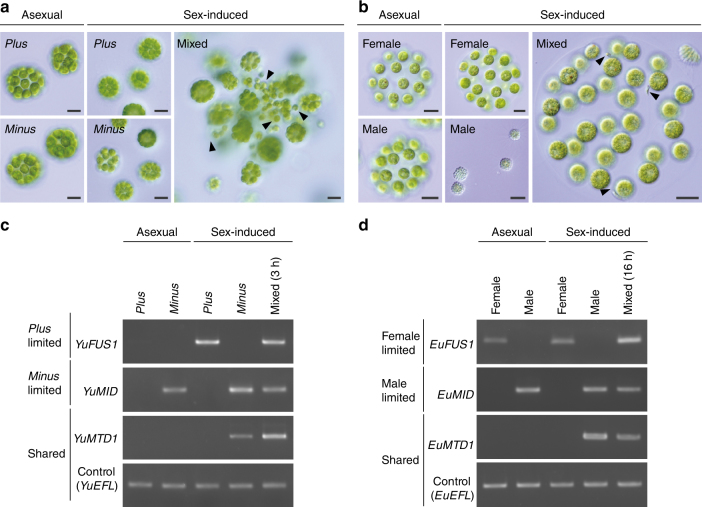
Sex induction and associated gene expression alternations in isogamous *Y*. *unicocca* and anisogamous *Eudorina* sp. **a**, **b** Asexual and sex-induced individuals of opposite sexes of *Y*. *unicocca* (*plus/minus*) (**a**) and *Eudorina* sp. (female/male) (**b**). Mating reactions (mixed, right panels) occurred after mixing induced cultures of the two sexes (middle panels). In *Y*. *unicocca* (**a**), clumping of the colonies and release of single-celled isogametes (arrowheads) were observed 1 h after mixing. In *Eudorina* sp. (**b**), sex induction treatment resulted in the formation of sperm packets and the packet dissociated into individual sperm that penetrated into a female colony (arrowheads) within 16 h after mixing. Scale bars, 20 µm. **c**, **d** Gene expression pattern of volvocine sex-limited genes in *Y*. *unicocca* (**c**) and *Eudorina* sp. (**d**). Semi-quantitative RT-PCR analyses were performed using the same cultures for **a** and **b**. All gels were run under the same experimental conditions^44^, and the cropped gel images are shown. Full-length gel images with size markers are presented in Supplementary Fig. 11

Since female gametes of *Eudorina* and isogametes of *Yamagishiella* have no prominent morphological differentiation from their respective vegetative cells (Fig. 3a, b)^18^, the emergence of anisogamy from isogamy in the *Yamagishiella*/*Eudorina*-like ancestor appears to have been based on acquisition of the ability to form small male gametes. The present genome comparison demonstrated that the only male-limited gene in *Eudorina MT* is *MID* (Fig. 2b). The *MID* gene is present only in the *minus*/male *MT* haplotypes of heterothallic volvocine organisms^28^ and serves as a master regulator for mating-type determination in isogamous *C*. *reinhardtii*^6^. Recent molecular genetic approach in oogamous *V*. *carteri* showed that transformation of a female strain with *MID* enables the eggs (female gametes) to produce sperm packets (bundles of male gametes) and that a *MID*-knocked down male strain produces androgonidia (male reproductive initials) that can function as eggs^10^. Therefore, although there are an additional eight male-limited and five female-limited *MT* genes in the expanded *MT* of *V*. *carteri*^9^ that likely play a role in gamete fitness and early sexual development, none of them other than *MID* appear to have an essential role for the dimorphic gamete differentiation^10^. It follows that the evolution of males in volvocine algae might have resulted from altered function of the sex-determining protein MID or its target genes.

The isogamous FUS1 protein is a single-pass transmembrane protein that is present on the mating structure of *plus* gametes and required to recognize and adhere to *minus* gametes^11, 29, 30^. Previously, *FUS1* homologs have been reported in isogamous *C*. *reinhardtii*^29, 30^ and *G*. *pectorale*^11^, but were not found in the oogamous *V*. *carteri* genome^9^, suggesting that *FUS1* was lost at some point after the *G*. *pectorale–V. carteri* lineages split^31^. Our data showed that isogamous *Y*. *unicocca* and anisogamous *Eudorina* sp. both had a *FUS1* homolog that was *plus*- or female-limited in *MT*, respectively (Fig. 2a, b; Supplementary Figs 4–8; Supplementary Note 1), and suggest that loss of *FUS1* occurred subsequent to the *Eudorina*–*V*. *carteri* split.

### Sex-related gene expressions in *Yamagishiella* and *Eudorina*

Expression programs of sex-related *MT* genes were evaluated for *Y*. *unicocca* and *Eudorina* sp. (Fig. 3). Unlike the case in *C*. *reinhardtii* and *G*. *pectorale*^6, 8^, but similar to *V*. *carteri*^9^, the *MID* mRNA was expressed constitutively in *Y*. *unicocca minus* cells and *Eudorina* sp. males. The gamete adhesion gene *FUS1* from *Y*. *unicocca plus* was expressed only in gametes as is also the case for *C*. *reinhardtii* and *G*. *pectorale FUS1*^11, 29^, but *Eudorina* sp. female *FUS1* was most strongly expressed after mixing female with male gametes. The *MTD1* gene in *C*. *reinhardtii* is found only in the *minus MT* haplotype, is expressed in gametes, and is required for efficient gametogenesis^32^, but appears to have lost its function in *V*. *carteri* where it is a pseudogene^9^. In *Y*. *unicocca* and *Eudorina* sp., *MTD1* is autosomal with a copy in both sexes (Fig. 1, Supplementary Table 2). However, *MTD1* expression in both *Eudorina* sp. and *Y*. *unicocca* was restricted to *minus*/male gametes (Fig. 3c, d), indicating a conserved function in gametogenesis in all genera except *Volvox* (Supplementary Fig. 9 and 10). The evolution of a highly reduced male *MT* in *Eudorina* sp. containing only the single male-limited *MID* gene might have been possible with minor changes that placed expression of *MTD1* under mating-type control allowing it to reside outside of the *MT* locus.

## Conclusions

We have illuminated here the initial transition of the sex-determining chromosomal region or *MT* during the evolution of anisogamy, and shown that anisogamy can evolve without increased *MT* complexity. Only two sex-limited genes that encode the sex-determining factor *MID* (*minus*/male) and the gamete recognition factor *FUS1* (*plus*/female) constitute the core of the *MT* haplotypes in both isogamous and anisogamous volvocine algae. Therefore, in this lineage the transition to anisogamy likely involved direct modification of the sex determination pathway controlled by *MID* rather than by acquisition of new gamete size control genes in *MT*. The presence of *FUS1* not only in isogamous but also in anisogamous volvocine algae suggests that both systems share the FUS1-dependent gamete recognition mechanism. Future investigation of sex-related genes and their roles in anisogamy and gamete dialogues in volvocine algae holds great promise for understanding the dynamics of mating system evolution in eukaryotes.

## Methods

### Strains

For *Yamagishiella*, *Y*. *unicocca* strains 2012-1026-YU-F2-6 (NIES-3982, mating-type *plus*) and 2012-1026-YU-F2-1 (NIES-3983, mating-type *minus*) were used throughout the study. For the whole-genome sequencing of *Eudorina*, *Eudorina* sp. strains 2010-623-F1-E4 (NIES-3984, female) and 2010-623-F1-E2 (NIES-3985, male) were used. In other *Eudorina* experiments, two other sibling strains of the former two, *Eudorina* sp. strains 2010-623-F1-E8 (NIES-4018, female) and 2010-623-F1-E3 (NIES-4100, male), were used unless otherwise stated.

### De novo whole genome assembly

Genomic DNAs were prepared according to the method of Miller et al.^33^. Whole-genome sequencing of *plus* and *minus* strains of *Y*. *unicocca* and male and female strains of *Eudorina* sp. were performed using PacBio and Illumina technologies as described previously^34^. Briefly, genomic DNA was sheared using a DNA shearing tube, g-TUBE (Covaris). Several 20 kb libraries for P5-C3 and P6-C4 sequencing were constructed and sequenced on SMRT cells in PacBio RS II (Pacific Biosciences). These reactions generated 2.5 M and 4.1 M sub reads (total bases: 17.2 Gb and 22.8 Gb, respectively) for *plus* and *minus* strains of *Y*. *unicocca*, respectively, and 2.6 M and 4.4 M sub reads (total bases: 15.6 Gb and 23.0 Gb, respectively) for male and female strains of *Eudorina* sp., respectively. Sequencing coverage was about 128x, 162x, 93x, and 125x based on the estimated genome size, respectively. In each of the four strains, PacBio reads were assembled de novo with HGAP3 assembler (Pacific Biosciences). Furthermore, genomic DNA was fragmented using a DNA Shearing System, S2 Focused-ultrasonicator (Covaris). Illumina paired-end libraries (insert sizes with 400 bp for *plus* and *minus* strains of *Y*. *unicocca*, 600 bp for male strain of *Eudorina* sp., and 400 bp for female strain of *Eudorina* sp.) were constructed using a TruSeq DNA Sample Prep Kit (Illumina) according to the manufacturer instructions. These libraries were sequenced using Illumina HiSeq 2000 and 2500 sequencers (230.5 M and 197.0 M reads with 150 bp read length for *plus* and *minus* strains of *Y*. *unicocca*, respectively, 251.2 M reads with 250 bp read length for male strain of *Eudorina* sp., and 165.3 M reads with 100 bp read length for female strain of *Eudorina* sp.). Total bases and sequencing coverage were 34.6 Gb (258x), 29.5 Gb (210x), 62.8 Gb (372x), and 16.5 Gb (89x), respectively. The Illumina data were then mapped against the PacBio assembly sequence using BWA-MEM Release 0.7.7^35^ including error correction with the samtools/bcftools/vcfutils.pl program v0.1.19 (https://samtools.sourceforge.net/), ultimately giving a set of nuclear genome sequence. We performed a long-range scaffolding using paired-end Sanger sequences from 38,400 and 3840 fosmid clones of female and male strains of *Eudorina* sp., respectively (DRA: DRA004920, DRA002727, and DRA004919; whole genome assembly: BDSI01000001-BDSI01003180, BDSJ01000001-BDSJ01002471, BDSK01000001-BDSK01001897, BDSL01000001-BDSL01001461).

### Sex-determining region identification

Candidate scaffolds for entire sex-determining regions (*Y*. *unicocca plus*: Scaffold0026/0199/0237; *minus*: Scaffold0005/0230/0253/0437/1431; *Eudorina* sp. Female: scaffold1024; male: scaffold1040) were screened as major significant matching subjects with more than three non-overlapping protein hits (cutoff maximum E-value: 1e−10^36^) by TBLASTN (NCBI) on de novo assemblies of *Y*. *unicocca* and *Eudorina* sp. with 80 proteins on *V*. *carteri* female *MT* (Genbank Acc. No. GU784915) as queries and then dotplot-analyzed between haplotypes of same species using YASS (https://bioinfo.lifl.fr/yass/index.php)^37^ to detect the rearranged genomic regions of *MT*.

### Gene identification

We performed TBLASTN searches against the genome assembly databases of *Y*. *unicocca* and *Eudorina* sp. with the volvocine sex-limited proteins (*Gonium pectorale* FUS1, BAU61607^11^; *G*. *pectorale* MTD1, BAI49487^38^) as the queries, retrieved sequences with the highest similarity, and designed gene-specific primers (listed in Supplementary Table 3) based on these sequences. To identify the ORF sequences, polyadenylated mRNAs from each sample were isolated using Dynabeads Oligo (dT)_25_ (Thermo Fisher Scientific), reverse transcribed with Superscript III reverse transcriptase (Thermo Fisher Scientific), and amplified with KOD FX Neo DNA polymerase (Toyobo) and the gene-specific primers. To obtain the full-length cDNA sequences, 5′RACE and 3′RACE were performed using the GeneRacer kit (Thermo Fisher Scientific). The PCR products were directly sequenced, or first cloned into the pCR4Blunt-TOPO vector (Thermo Fisher Scientific) and then sequenced, using an ABI PRISM 3100 Genetic Analyzer (Thermo Fisher Scientific) with a BigDye Terminator cycle sequencing ready reaction kit, v.3.1 (Thermo Fisher Scientific). Full-length *MID* genes of *Y*. *unicocca* (*minus* strain NIES-1859) and *Eudorina* sp. (male strain NIES-2735) were determined using the degenerate PCR method^7, 8^.

Other gene models on *MT* scaffolds were predicted by Augustus^39^ with the *C*. *reinhardtii* parameter and then manually curated based on the similarity among *C*. *reinhardtii* and *V*. *carteri* gene models (JGI).

*MT* genome sequences harboring rearranged domains with sex-limited genes and gametologs in *Y*. *unicocca* and *Eudorina* sp. and the autosomal gene *YuMTD1* are available under accession numbers LC314412–LC314416.

### Preparation of asexual and sex-induced samples of *Yamagishiella* and *Eudorina*

Asexual samples of *Y*. *unicocca* were obtained by culturing the algae in screw-cap tubes (18 × 150 mm) containing ~11 ml AF-6 medium^40, 41^, on a 14-h light/10-h dark cycle (light intensity: 60–110 μmol m^−2^ s^−1^) at 23 °C for 3–5 days. To induce sexual reproduction of *Y*. *unicocca*, asexually growing algae (~0.2 ml) were inoculated into 11 ml “VTAC + soil extract medium” (VTAC medium^41, 42^ supplemented with 3%(v/v) soil extract (~0.5 mg of paddy soil suspended in 20 ml distilled water and autoclaved for 10 min)) in a tube, and cultured for 8 days under the same condition. The algal culture of each sex was then transferred into Petri dishes (60 mm × 15 mm), incubated for further 4 days, and used as a sex-induced *Y*. *unicocca* sample for the following analysis. “Mixed” sample of *Y*. *unicocca* were obtained by mixing the sex-induced samples (1 ml each) of both mating types in Petri dishes (30 mm × 10 mm), which were subsequently incubated for 3 h under the same condition, and used for semiquantitative RT-PCR.

Asexual samples of *Eudorina* sp. were obtained by culturing the algae in the screw-cap tubes containing ~10 ml SVM medium^43^, on a 14-h light/10-h dark cycle (light intensity: 180–320 μmol m^−2^ s^−1^) at 25 °C for 3 days. To induce sexual reproduction of *Eudorina* sp., ~0.4 mL of asexually grown algae in SVM medium were inoculated into Petri dishes (60 mm × 15 mm) containing ~11 ml “VTAC + soil extract medium” and cultured for 3 days under the same condition. The 11 ml culture of each sex was then transferred into Petri dishes (90 mm × 20 mm), diluted with twice volume of mating medium^42^, and cultured for further 8 h under the same condition to form a sex-induced male or female culture of *Eudorina* sp., in which formation of sperm packets was observed in the male strain (Fig. 3b). “Mixed” samples of *Eudorina* sp. were prepared by mixing the sex-induced female and male samples (5 ml each) in Petri dishes (60 mm × 15 mm), subsequently incubated for 16 h under the same condition, and used for the following analysis.

All microscopic images were acquired using a BX53 microscope (Olympus) equipped with differential interference contrast optics. The digital images were captured using a DP71 camera (Olympus) with DP controller software (Olympus), and their levels were adjusted with Adobe Photoshop CS6 (Adobe Systems Inc.).

### Semiquantitative RT-PCR analysis

From *Y*. *unicocca* samples, polyadenylated mRNAs were isolated using Dynabeads Oligo (dT)_25_ and reverse transcribed as described above. From *Eudorina* samples, total RNAs were extracted with TRI reagent (Molecular Research Center), treated with DNase I (amplification grade; Thermo Fisher Scientific), and reverse transcribed with Superscript III reverse transcriptase and Oligo (dT)_20_ primer (Thermo Fisher Scientific). PCR reactions were performed with KOD FX Neo DNA polymerase (Toyobo). Primer sequences and PCR condition are listed in Supplementary Table 3 online. Under the conditions, all primer sets produced amplicons of the expected size and sequence (confirmed by direct sequencing). The PCR products were electrophoresed on 2% (w/v) agarose gels and stained with ethidium bromide^44^. The gel images were captured using a ChemiDoc XRS system (Bio-Rad) with Quantity One software (Bio-Rad) and their levels were adjusted as described above.

### Molecular evolutionary analysis

Divergence scores of synonymous and non-synonymous substitutions between gametologs were computed using yn00 of the PAML4 package^45^; nonsynonymous and synonymous site divergence of aligned coding sequences of gametologs was calculated based on Yang and Nielsen^46^ with equal weighting between pathways, and the same codon frequency for all pairs^11^.

### Data availability

Raw reads, genome assemblies, and annotations were deposited at DDBJ/EMBL/GenBank under the accessions as follows; DRA: DRA004920, DRA002727, and DRA004919; whole genome assembly: BDSI01000001-BDSI01003180, BDSJ01000001-BDSJ01002471, BDSK01000001-BDSK01001897, BDSL01000001-BDSL01001461; annotations: LC314412- LC314416. All the other data generated or analyzed during this study are included in this published article and its Supplementary information.

## Electronic supplementary material

Supplementary Information

Description of Additional Supplementary Files

Supplementary Data 1
