# Supplementary material for: Anisogamy evolved with a reduced sex-determining region in volvocine green algae
Source: Commun Biol. 2018 Mar 8;1:17. doi: 10.1038/s42003-018-0019-5 (PMC6123790; doi:10.1038/s42003-018-0019-5)
Supplement: Supplementary file 1 — Supplementary Information [file 42003_2018_19_MOESM1_ESM.pdf]

Supplementary Table 1. Comparison of whole genome and *MT* properties of the volvocine algae. n.d., not determined.

	Sexual reproduction	Isogamy						Anisogamy		Oogamy	
	Species	*Chlamydomonas reinhardtii* [a]		*Gonium pectorale* [a]		*Yamagishiella unicocca*		*Eudorina* sp.		*Volvox carteri* [a]	
	Mating type/ sex	*Plus*	*Minus*	*Plus*	*Minus*	*Plus*	*Minus*	Female	Male	Female	Male
Whole genome	Size/total length (Mb)	111.1	n.d.	n.d.	148.8	134.2	140.8	184.0	168.6	131.1	n.d.
	%GC	64.1	n.d.	n.d.	64.5	61.1	60.8	61.0	61.3	56.1	n.d.
MT (R domain[b])	Size (Mb)	0.310	0.204	0.366	0.499	0.268	0.165	0.090	0.007	1.51	1.13
	%GC	60	61	59.7	61.0	60.1	60.3	53.9	51.4	52	53

[a]Based on Hamaji *et al* [1].

[b]Sizes for rearranged (R) regions are indicated.

Supplementary Table 2. Presence/absence of mating type/sex-specific *MT* gene homologs in mating haplotypes of five volvocine species. Gene presence or absence (-) were identified mainly from TBLASTN.

	Isogamy			Anisogamy	Oogamy
Gene name/ query	*Chlamydomonas reinhardtii* DDBJ/EMBL/ Genbank GU814014-5	*Gonium pectorale* DDBJ/EMBL/ Genbank LC062718-9	*Yamagishiella unicocca* (this study)	*Eudorina* sp. (this study)	*Volvox carteri* DDBJ/EMBL/ Genbank GU784915-6
MID	*Minus*	*Minus*	*Minus*	Male	Male
MTD1	*Minus*	*Minus*	Both/*MT*-linked	Both/*MT*-linked	Male (pseudo)
FUS1	*Plus*	*Plus*	*Plus*	Female	-
CrMTA1	*Plus*	-	-	-	-
CrEZY2	*Plus*	-	-	-	-
VcFSI1f	-	-	Both/autosomal	Both/autosomal	Female
VcHMG1f	-	-	-	Both/autosomal	Female
VcMTF0821	-	-	-	-	Female
VcMTF0991	-	-	-	-	Female
VcMTF2030	-	-	-	-	Female
VcMTM0097	-	-	Both (partial)/ autosomal	Both/autosomal	Male
VcMTM0441	-	-	-	-	Male
VcMTM0564	-	-	-	-	Male
VcMTM0665	-	-	-	-	Male
VcMTM0761	-	-	-	-	Male
VcMTM0832	-	-	-	-	Male
VcMTM0897	-	-	-	-	Male
VcMTM0946	-	-	-	-	Male

Supplementary Table 3. Primer sequences, PCR conditions and product sizes. PCR reactions were performed with the following condition unless otherwise described in footnotes: 94°C for 2 min, followed by the indicated number of two-step cycles of 98°C for 10 sec and 68°C for the indicated extension time.

Primer name	Primer sequence (5' to 3')	Cycle numbers	Extention time	Product size (bp)
Semi-quantitative RT-PCR of *Y. unicocca* (Fig. 3c)				
YuFUS1_F14	ACAACACTTTCGTCTTCCAAAGAAGC	35	30 sec	608
YuFUS1_R2	GTGAAATCTCCGGCCTGATCTGAG			
YuMID_F1	TTCCACGCGCACATTCAAGGCTCTG	28	30 sec	352
YuMID_R1	GGCTGGCAACCTTTCGATATGGCCA			
YuMTD1_F1	GGCTACGTCTCATTCCCGCAGTC	28	30 sec	632
YuMTD1_R2	CTCTGCGCCAAGAACATCGTCAC			
YuEFL_F1	AGAAGGAGCGCTACGACGAGATTGC	22	30 sec	403
YuEFL_R2	GTGCAAGGGTTAGCGGTGGTGTG			
Semi-quantitative RT-PCR of *Eudorina* sp. (Fig. 3d)				
EuFUS1_F8	CACTGCACTTTCCTTGGTTCTTGC	38	30 sec	323
EuFUS1_R12	GCTGGTATGACGCCCTGATTGTC			
EuMID_F2	TGGACAAGTCACACTGGAGCTGCAT	28	30 sec	341
EuMID_R1	CCATCGTGGGATTCCTAGCTGCCTG			
EuMTD1_F1	AGCATGACAACGCTCGCATGGTGG	28	30 sec	409
EuMTD1_R1	GTCACCAGCACCCTCTCCGCCAG			
EudEF1A_F1	CTTGTCGACACGCTTGTGGTG	22 (3-step) [a]	30 sec	451
EudEF1A_R2	CAAGAAGGAGCGCTACGATGAGAT			

ORF confirmation				
YuFUS1_F15	AATGTTTCATGTATGTTTAGCGATTG	35 (3-step) [a]	1.5 min	2,700
YuFUS1_R10	CCCGTGCATCAATATACGTCATTC			
YuMTD1_F5	AGCATCGCACCTCACTCGTCCGTC	28	1 min	2,107
YuMTD1_R1	TCAGCATCCTCGCCCACCATGC			
EuFUS1_F19	GAATAGACCACACTTTGTTGCA	35 (3-step) [a]	1.5 min	2,601
EuFUS1_R17	CTGGAAGTCAATGCAATATGGC			
EuMTD1_F9	TGCTGGTCACTGGTGGTGCAAATCG	28	1 min	1,969
EuMTD1_R6	GGGCTACTCCATCCTGTCCATCCACC			
5'RACE				
YuFUS1_R8	CCGAATATGAGGCCCAGGTTCACA	35 (step-down) [b]	40 sec	281 + adapter
YuMTD1_5GS2	GCCGCGCCACAGCGAAATCGACAG	35 (step-down) [b]	30 sec	626 + adapter
EuFUS1_R15	AGGCTCCAACGTGAAGACGTAAGG	35 (step-down) [b]	40 sec	209 + adapter
EuMTD1_5GS3	GTCAGCTCCCACAGTCGCATCACTGCT	35 (step-down) [b]	30 sec	452 + adapter
3' RACE				
YuFUS1_F11	CAACATGCCTGACACGGTGAAGC	35 (step-down) [b]	40 sec	>1,378<br>>377 [c]
YuFUS1_F10	CAAATTGCTGCGCTGACCATTGG	35 (step-down) [b]	40 sec	>1,341<br>>340 [c]
YuMTD1_F6	GTGACGATGTTCTTGGCGCAGAG	35 (step-down) [b]	30 sec	>776
YuMTD1_F7	GCCCTTCTTGCGCGATGTTTGAGA	35 (step-down) [b]	30 sec	>646
EuFUS1_F16	TCGACTCCATCACAAGCTGTCCTC	35 (step-down) [b]	1 min	>1,548
EuFUS1_F10	TCCTACATGAACATTGTGCAGATACCT	20 (second [nested] PCR)	1 min	>1,467
EuMTD1_F7	GGTGGATGGACAGGATGGAGTAGC	35 (step-down) [b]	30 sec	>694
EuMTD1_F11	TACAGGTACCAGCATCCGTTGTGT	35 (step-down) [b]	30 sec	>174

[a] Three-step PCR were performed as follows: 94°C for 2 min, followed by the indicated number of cycles of 98°C for 10 sec, 65°C (for *EuEFL*), 63°C (*YuFUS1* ORF) or 62°C (*EuFUS1* ORF) for 30 sec, and 68°C for the indicated extension time.

[b] Step-down PCR were performed with the indicated total number of cycles and extension time according to the manufacturer's instruction.

[c] PCR products arose from alternative polyadenylation were detected.

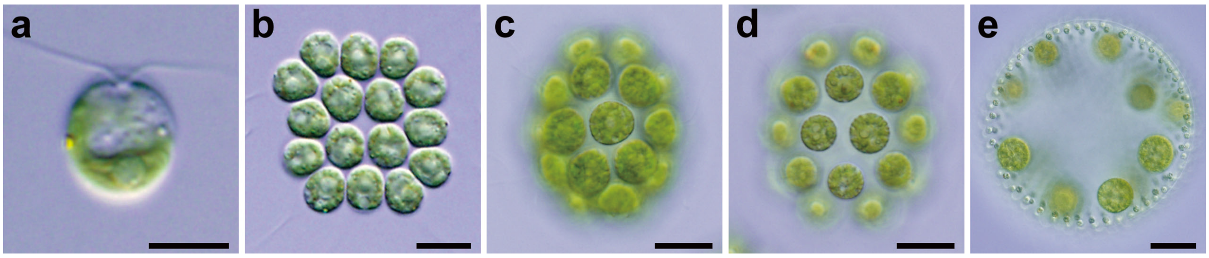

Supplementary Figure 1. Vegetative morphologies of five volvocine algae. (**a**) *Chlamydomonas reinhardtii* (C-239, mating-type *plus*). Scale bar, 5 µm. (**b**) *Gonium pectorale* (K41, mating-type *plus*). Scale bar, 10 µm. (**c**) *Yamagishiella unicocca* (2012-1026-YU-F2-6, mating-type *plus*). Scale bar, 20 µm. (**d**) *Eudorina* sp. (2010-623-F1-E8, female). Scale bar, 20 µm. (**e**) *Volvox carteri* (Eve10, female). Scale bar, 50 µm. All images were obtained as described for Fig. 3a,b in the main text (see the Methods section of the main text).

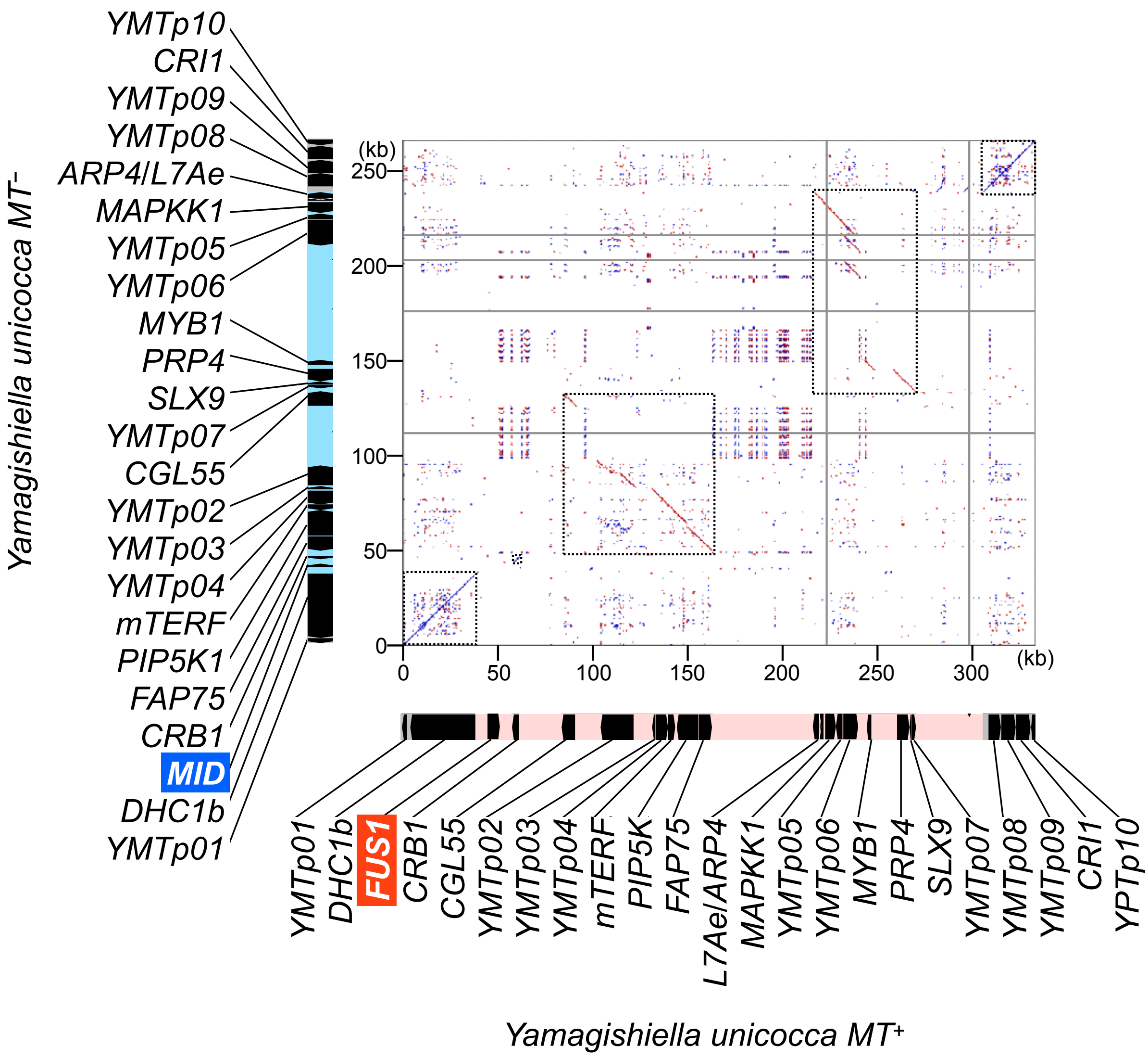

Supplementary Figure 2. *Yamagishiella unicocca* dotplot between *minus* (vertical) and *plus* (horizontal) specific regions (pale blue and pale red, respectively) and parts of flanking regions (gray boxes). Borders of scaffolds based on the *de novo* whole genome assembly are depicted as gray lines in the plot. Alignable region blocs between two mating types are marked with dotted boxes.

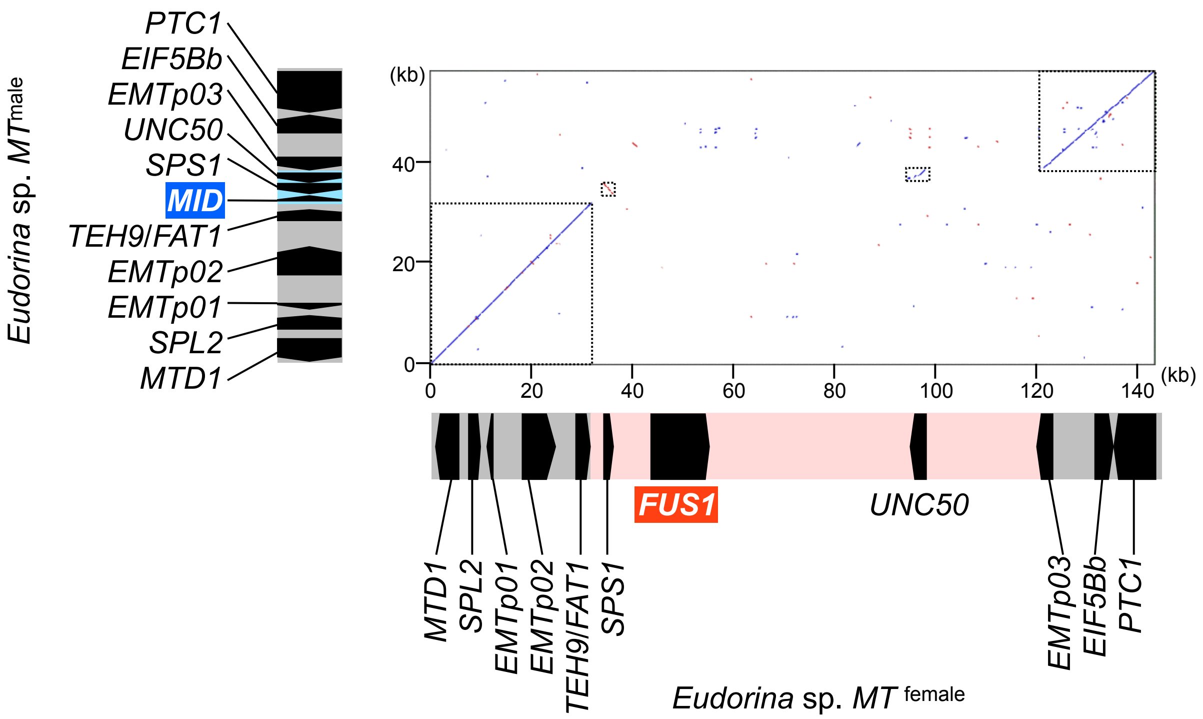

Supplementary Figure 3. *Eudorina* sp. dotplot between male (vertical) and female (horizontal) specific regions (pale blue and pale red, respectively) and parts of flanking regions (gray boxes). Alignable region blocs between two mating types are marked with dotted boxes.

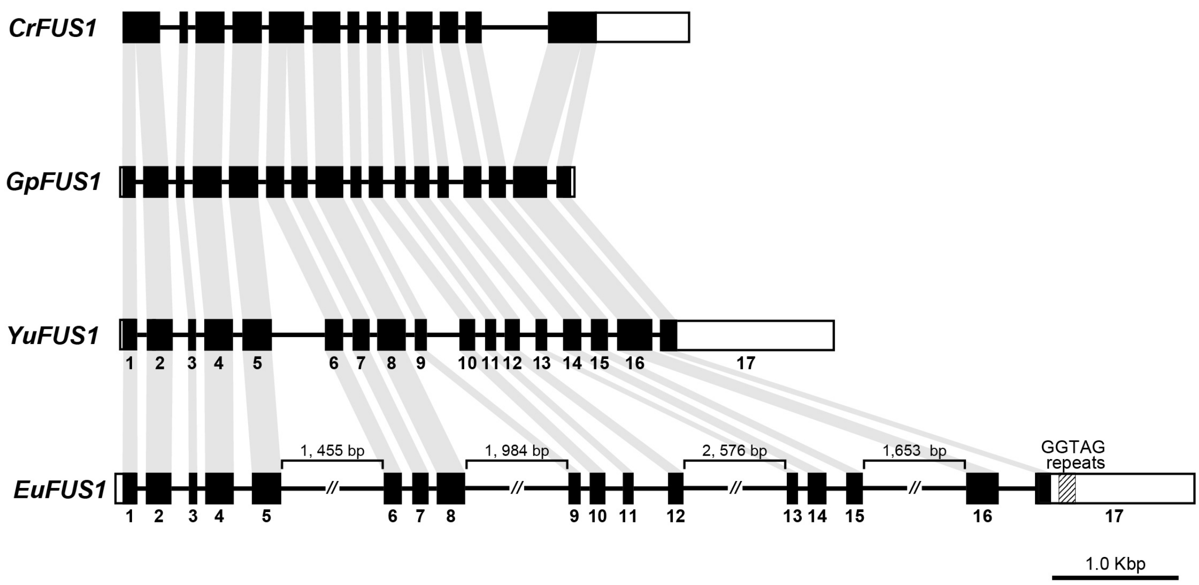

Supplementary Figure 4. Structures of the volvocine *FUS1* genes. The exon-intron structures of the *FUS1* genes from *C. reinhardtii* (*CrFUS1* [2]), *G. pectorale* (*GpFUS1* [1]), *Y. unicocca* (*YuFUS1,* identified in this study), and *Eudorina* sp. (*EuFUS1*, identified in this study) are shown for comparison. Filled and open boxes represent coding and non-coding exon sequences, respectively. Numbers below boxes for *YuFUS1* and *EuFUS1* indicate exon numbers. For the non-coding regions of *YuFUS1* and *EuFUS1*, the longest sequence detected by the RACE analyses is depicted. Lines between boxes represent introns. Gray boxes link homologous coding sequences. In the 3' non-coding region of *EuFUS1*, a short tandem repeat of 5 bp ("GGTAG", s hatched box) was present. The GGTAG repeat number in the *EuFUS1* cDNA is up to 27 (same as in the genomic sequence), although fewer repeats (6 and 11) were also detected by the RACE analysis.

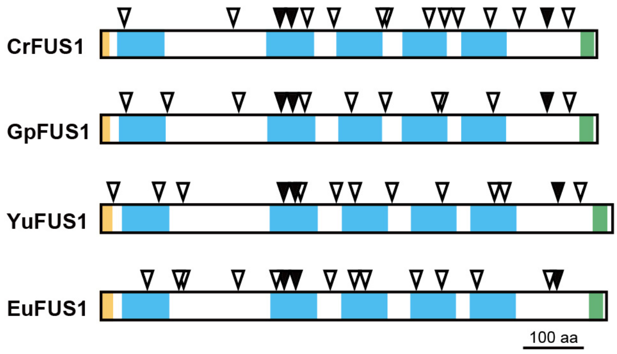

Supplementary Figure 5. Schematic representations of volvocine FUS1 proteins. Orange, blue and green bars represent signal peptide, Early immunoglobulin (Ig)-like repeat that we term the "FUS1" domain here, and transmembrane domain, respectively. Potential N-glycosylation sites conserved in all four proteins are indicated with solid arrowheads. Other potential N-glycosylation sites are indicated with open arrowheads. See Supplementary Figs. 6 and 7 for details.

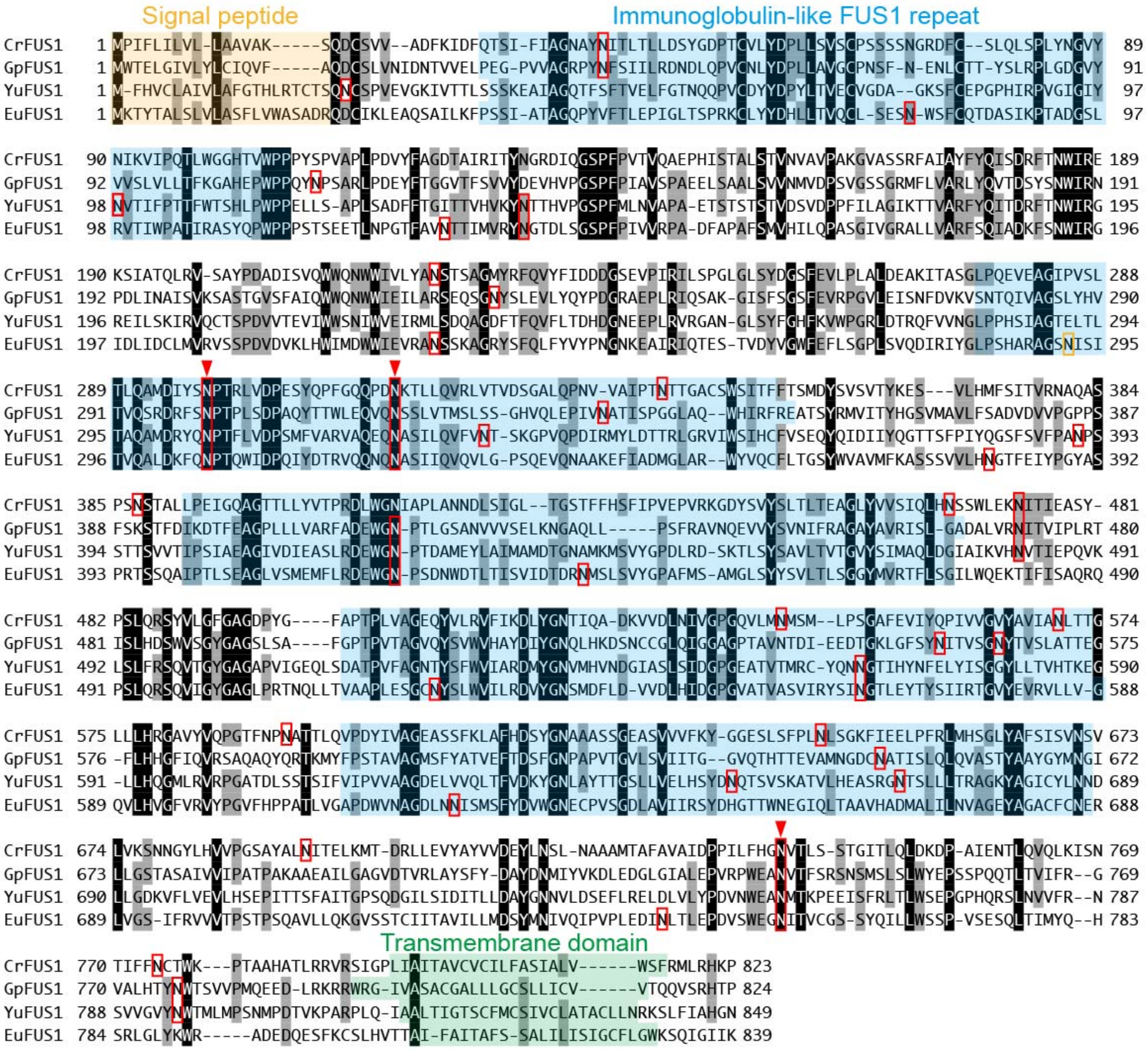

Supplementary Figure 6. Alignment of the deduced FUS1 amino acid sequences. The FUS1 sequences of *C. reinhardtii* (CrFUS1, accession number AAC49416), *G. pectorale* (GpFUS1, accession number BAU61607), *Y. unicocca* (YuFUS1, identified in this study) and *Eudorina* sp. (EuFUS1, identified in this study) were aligned using the MUSCLE program built in the GENETYX-MAC software ver. 17.0.6 (Genetyx Co.) with manual adjustments. Residues identical in all four sequences are shaded in black, and residues identical in three sequences are shaded in gray. The putative signal peptide and transmembrane domain in each sequence were predicted by Phobius (http://phobius.sbc.su.se) [3] and shaded in orange and green, respectively. The immunoglobulin-like FUS1 repeats are shaded in blue (see Supplementary Note 1 for details). Potential N-glycosylation sites were predicted by GlycoEP (http://www.imtech.res.in/raghava/glycoep/) [4] [using the Binary Profile of Pattern (BPP) methods with the default support vector machine threshold of 0.0.] and marked with red boxes. Red arrowheads above the boxes indicate the N-glycosylation sites conserved in all four proteins.

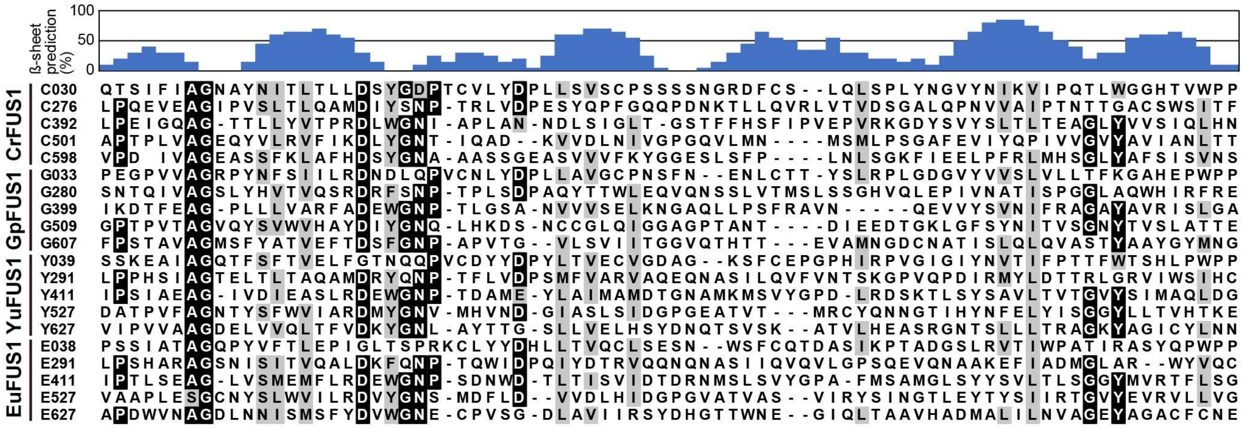

Supplementary Figure 7. Alignment of the immunoglobulin-like FUS1 repeats. The alignment was manually edited basically according to the previous studies [1,5]. The number (with an alphabet) at the left of each sequence indicates the position of the first residue of each repeat within the respective FUS1 sequence. Residues identical or similar in more than 50% of the sequences are shaded black or gray, respectively. Blue bars on the top of the alignment indicate the percentages of beta-sheet forming amino acids [predicted by GOR secondary structure prediction method [6] using the GENETYX-MAC software ver. 17.0.6 (Genetyx Co.)] in all amino acids.

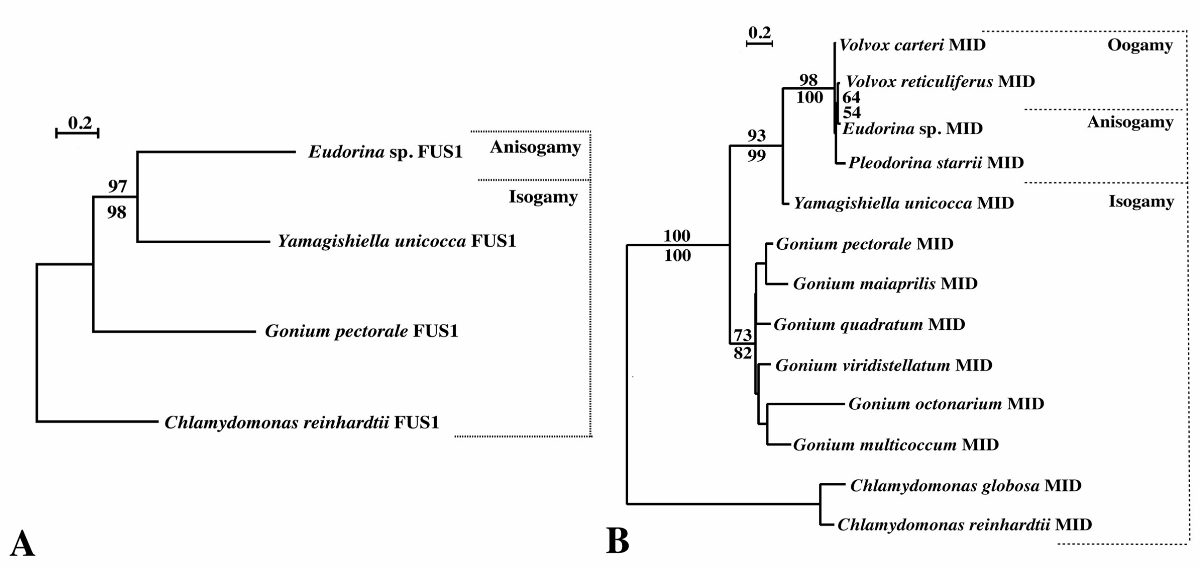

Supplementary Figure 8. Phylogeny of two conserved sex-limited genes in the volvocine lineage. The evolutionary history was inferred based on the deduced amino acid sequences by maximum likelihood (ML) method using the models selected by MEGA7 [7]. Branch lengths are proportional to the evolutionary distances indicated by the scales above the tress. Numbers above and below branches indicate bootstrap values [8] of 1000 replications based on ML and neighbor-joining (NJ) [9] methods, respectively, by using MEGA7.

(**A**). Female- or mating type *plus*-specific FUS1 homologs from four species. ML analysis was performed by WAG [10]+I+F model using the alignments in Supplementary Fig. 6. NJ bootstrap analysis was carried out by JTT distance [11]. All positions containing gaps and missing data were eliminated. There were a total of 787 positions in the final dataset.

(**B**). Male-specific or mating type *minus*-specific MID homologs from 13 heterothallic species. ML and NJ analyses were performed by JTT+G model using the alignment composed of only heterothallic species studied in this study and previously [12]. All positions containing gaps and missing data were used.

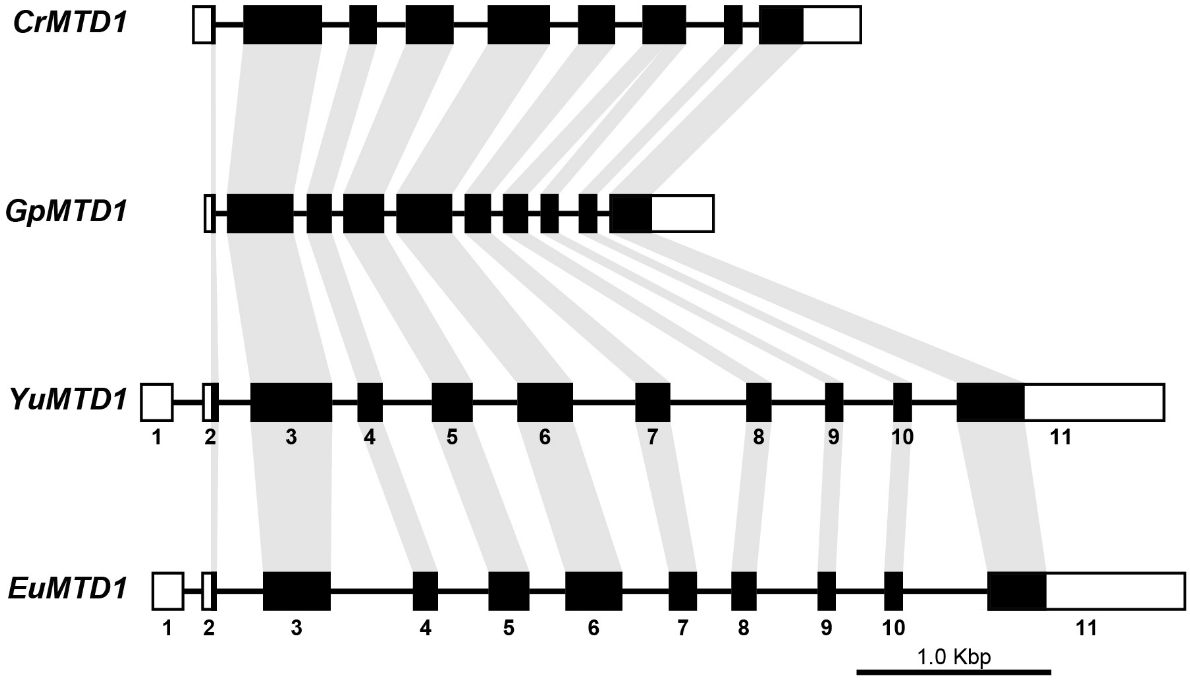

Supplementary Figure 9. Structures of the volvocine *MTD1* genes. The exon-intron structures of the *MTD1* genes from *C. reinhardtii* (*CrMTD1* [13]), *G. pectorale* (*GpMTD1* [14]), *Y. unicocca* (*YuMTD1,* identified in this study), and *Eudorina* sp. (*EuMTD1*, identified in this study) are shown. Exons, introns and other sequence features are shown in the same manner as Supplementary Fig. 4. For the non-coding regions of *YuMTD1* and *EuMTD1*, the longest sequence detected by the RACE analyses is depicted. The *MTD1* exon-intron structure is mostly conserved, although an additional splicing site is found in the 5’ untranslated region of *YuMTD1* and *EuMTD1*.

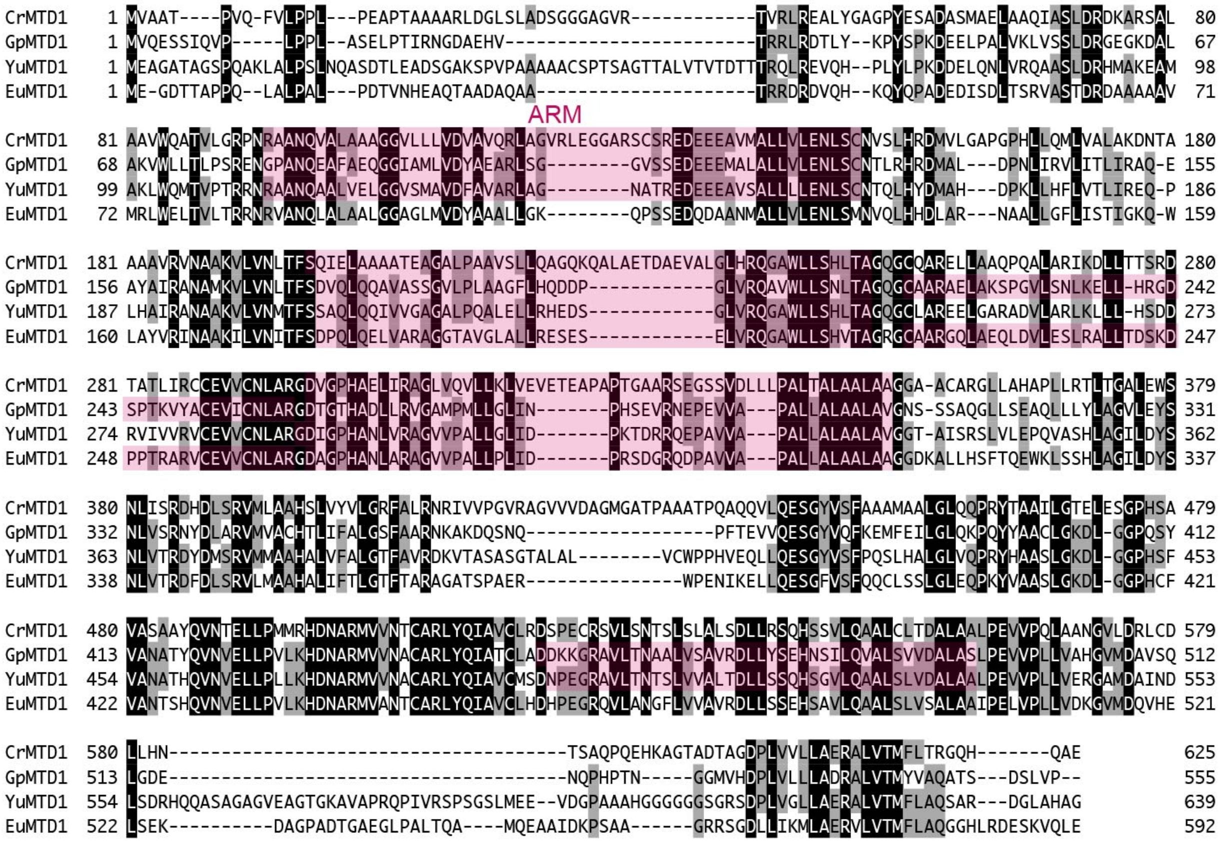

Supplementary Figure 10. Alignment of the deduced MTD1 amino acid sequences. The MTD1 sequences of *C. reinhardtii* (CrMTD1, accession number AAL14635), *G. pectorale* (GpMTD1, accession number BAI49487), *Y. unicocca* (YuMTD1, identified in this study) and *Eudorina* sp. (EuMTD1, identified in this study) were aligned using the MUSCLE program built in the GENETYX-MAC software ver. 17.0.6 (Genetyx Co.) with manual adjustments. Identical residues are shaded as described for Supplementary Fig. 6. The ARM (armadillo/beta-catenin-like repeats) domains were predicted by the genomic mode of the SMART database (http://smart.embl-heidelberg.de.) [15] (5 in GpMTD1; 4 in YuMTD1; 3 in CrMTD1 and EuMTD1) and shaded in magenta. In the volvocine algae as a whole, no transmembrane domain and signal peptide was confidentially predicted, suggesting that the MTD1 proteins are neither a membrane protein nor a glycoprotein.

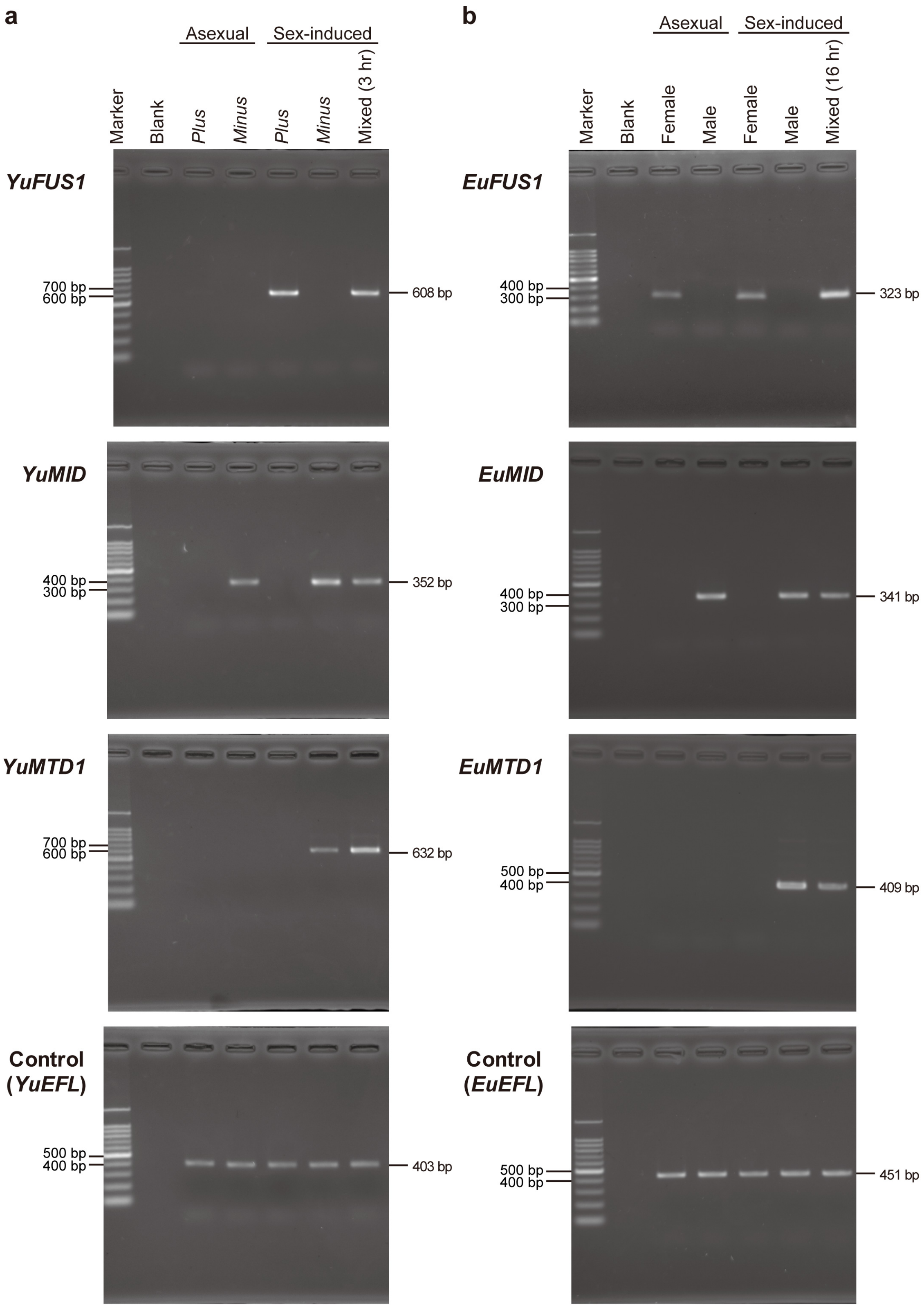

Supplementary Figure 11. Full-length gel images of semi-quantitative RT-PCR analyses in *Y. unicocca* (**a**) (associated to Fig. 3c) and *Eudorina* sp. (**b**) (associated to Fig. 3d). A 100-bp DNA ladder marker (Toyobo) was used as a molecular size marker (lane "Marker").

Supplementary Note 1. Structural conservation of volvocine FUS1s.

In this study, we identified homologs of *FUS1,* a volvocine isogametic adhesion factor-encoding gene [1,2,5]*,* from isogamous *Y. unicocca* and anisogamous *Eudorina* sp (Supplementary Figs. 4-8). It enabled us to define the structural features of the *FUS1* genes and their encoded-proteins in comparison with previously reported *FUS1s* from *C. reinhardtii* [2,5] and *G. pectorale* [1]. The *FUS1* exon-intron structure is well conserved among the volvocine species (Supplementary Fig. 4), with additional splicing variants detected for *Eudorina* sp. *FUS1* (*EuFUS1*). The *FUS1* genomic sequence of *EuFUS1* is remarkably long (12,760 bp) with four elongated introns, as compared to that of isogamous species (for example, the *YuFUS1* genomic sequence is 5,629 bp). The fact that not only isogamous volvocines but also anisogamous *Eudorina* sp. have FUS1 homologs suggest the conservation of sex recognition mechanisms in anisogamy.

Previous studies reported that FUS1 in *C. reinhardtii* and *G. pectorale* possess five internal ~90 amino acid repeats of an invasin/intimin immunoglobulin (Ig)-like sequence [1,5]. *C. reinhardtii* FUS1 was also predicted to contain an Ig-like filamin repeat in N-terminal region [16]. With the newly identified FUS1s from *Y. unicocca* and *Eudorina* sp., we reexamined the FUS1 domain structure. All four members of the volvocine FUS1s contain five weakly conserved repeated sequences (Supplementary Figs. 5 and 6), which are consistent with the previously defined repeats [1,5]. Some of these repeat (G033, Y039, Y527 and E038 in Supplementary Fig. 7) showed modest similarity to the SCOP superfamily “E set domains” ("Early" Ig-like fold families possibly related to the immunoglobulin and/or fibronectin type III superfamilies), on the Superfamily database 1.75 database (http://supfam.org/SUPERFAMILY/index.html) [17], rather than the invasin/intimin cell adhesion fragments superfamily. Among the superfamily “E set domains” members, filamin repeat (rod domain) family that contains F-actin cross-linking gelation factor (ABP-120) repeats from *Dictyostelium discoideum* is the most closely related to the internal FUS1 repeats. The three-dimensional structure of the ABP-120 repeat has been determined by NMR spectroscopy and shown to consist of seven β-sheets arranged in an Ig-like fold [18]. The internal FUS1 repeats are also predicted to form β-sheets (Supplementary Fig. 7). Therefore, we propose to define the internal FUS1 repeats as a “FUS1 domain” that has weak similarity to Ig-like filamin domain and also has the possibility to form an Ig-like structure that may be involved in gamete adhesion [19]. In addition, as described for *C. reinhardtii* FUS1 [2], volvocine FUS1s are also likely to be glycoproteins. Each FUS1 protein has 13 to 15 potential N-glycosylation sites among which three are conserved in all four FUS1s (Supplementary Figs.

5 and 6). Therefore, it is possible that sugar chains on FUS1 polypeptides play important and conserved role for sexual recognition in both isogamous and anisogamous system.

Supplementary References

1. Hamaji, T. *et al.* Sequence of the *Gonium pectorale* mating locus reveals a complex and dynamic history of changes in volvocine algal mating haplotypes. *G3 (Bethesda)* **6**, 1179–1189; 10.1534/g3.115.026229 (2016).

2. Ferris, P.J., Woessner, J.P. & Goodenough U.W. A sex recognition glycoprotein is encoded by the *plus* mating-type gene *fus1* of *Chlamydomonas reinhardtii. Mol. Biol. Cell* **7**, 1235–1248 (1996).

3. Käll, L., Krogh, A. & Sonnhammer, E. L. L. Advantages of combined transmembrane topology and signal peptide prediction–the Phobius web server. *Nucleic Acids Res.* **35,** W429–W432 (2007).

4. Chauhan, J. S., Rao, A. & Raghava, G. P. S. In silico platform for prediction of N-, O- and C-glycosites in eukaryotic protein sequences. *PLoS ONE* **8,** e67008; 10.1371/journal.pone.0067008 (2013).

5. Misamore, M.J. Gupta, S., & Snell, W.J. The *Chlamydomonas* Fus1 protein is present on the mating type *plus* fusion organelle and required for a critical membrane adhesion event during fusion with *minus* gametes. *Mol. Biol. Cell* **14**, 2530–2542 (2003).

6. Garnier, J., Osguthorpe D.J. & Robson, B. Analysis of the accuracy and implications of simple methods for predicting the secondary structure of globular proteins. *J. Mol. Biol.* **120**, 97–120 (1978).

7. Kumar, S., Stecher, G. & Tamura, K. MEGA7: Molecular Evolutionary Genetics Analysis version 7.0 for bigger datasets. *Mol. Biol. Evol* **33**, 1870–1874 (2016).

8. Felsenstein, J. Confidence limits on phylogenies: an approach using the bootstrap. *Evolution* **39**, 783–791(1985).

9. Saitou, N. & Nei, M. The neighbor-joining method: a new method for reconstructing phylogenetic trees. *Mol. Biol. Evol.* **4**, 406–425 (1987).

10. Whelan, S. & Goldman, N. A general empirical model of protein evolution derived from multiple protein families using a maximum-likelihood approach. *Mol. Biol. Evol.* **18**, 691–699 (2001).

11. Jones, D.T., Taylor, W.R. & Thornton, J.M. The rapid generation of mutation data matrices from protein sequences. *Comput. Appl. Biosci.* **8**, 275–282 (1992).

12. Yamamoto, K. *et al*. Molecular evolutionary analysis of a gender-limited *MID* ortholog from the homothallic species *Volvox africanu*s with male and monoecious spheroids. *PLoS ONE* **12**, e0180313; 10.1371/journal.pone.0180313 (2017).

13. Ferris, P.J., Armbrust, E.V. & Goodenough, U.W. Genetic structure of the mating-type locus of *Chlamydomonas reinhardtii. Genetics* **160**, 181–200 (2002).

14. Hamaji, T., Ferris, P. J., Nishii, I. & Nozaki, H. Identification of the *minus* mating-type specific gene *MTD1* from *Gonium pectorale* (Volvocales, Chlorophyta). *J. Phycol.* **45,** 1310–1314 (2009).

15. Letunic, I., Doerks, T. & Bork, P. SMART: recent updates, new developments and status in 2015. *Nucleic Acids Res.* **43**, D257–D260 (2014).

16. Mori, T., Igawa, T., Tamiya, G., Miyagishima, S. & Berger F. Gamete attachment requires GEX2 for successful fertilization in *Arabidopsis. Curr. Biol.* **24**, 170–175 (2014).

17. Oates, M. E. *et al.* The SUPERFAMILY 1.75 database in 2014: a doubling of data. *Nucleic Acids Res.* **43**, D227–33 (2015).

18. Fucini, P., Renner, C., Herberhold, C., Noegel, A.A. & Holak, T.A. The repeating segments of the F-actin cross-linking gelation factor (ABP-120) have an immunoglobulin-like fold. *Nat. Struct. Mol. Biol.* **4**, 223–230 (1997).

19. Mori, T., Kawai-Toyooka, H., Igawa, T. & Nozaki, H. Gamete dialogs in green lineages. *Mol. Plant* **8**, 1442–1454; 10.1016/j.molp.2015.06.008 (2015).
