# Supplementary material for: Anisogamy evolved with a reduced sex-determining region in volvocine green algae
Source: Commun Biol. 2018 Mar 8;1:17. doi: 10.1038/s42003-018-0019-5 (PMC6123790; doi:10.1038/s42003-018-0019-5)
Supplement: Supplementary file 2 — Description of Additional Supplementary Files [file 42003_2018_19_MOESM2_ESM.docx]

**Description of Additional Supplementary File**

File Name: Supplementary Data 1

Description: Presence/absence of homologs of gametologs in volvocine algae. Filled and gray-shaded cells indicate *MT* (formerly called as rearranged domains) and *MT* -linked (formerly called as telomere-oriented “T” or centromere-oriented “C” domains; established linkages in either of haplotypes in one species) genes found in both haplotypes (gametologs) of five volvocine species. Gene presence (scaffold information) or absence (N/D) were identified by TBLASTN. The “e” bloc in “*MT* scaffold” of *Gonium* is designated as “*MT* -linked” and autosomal ^1^ .

1 Hamaji, T. *et al*. Sequence of the *Gonium pectorale* mating locus reveals a complex and dynamic history of changes in volvocine algal mating haplotypes. *G3 (Bethesda)* 6, 1179–1189; 10.1534/g3.115.026229 (2016).
